# Perioperative mortality for radical cystectomy in the modern Era: experience from a tertiary referral center

**DOI:** 10.1590/S1677-5538.IBJU.2022.0405

**Published:** 2023-03-31

**Authors:** Sina Sobhani, Alireza Ghoreifi, Antoin Douglawi, Hamed Ahmadi, Gus Miranda, Jie Cai, Monish Aron, Anne Schuckman, Mihir Desai, Inderbir Gill, Siamak Daneshmand, Hooman Djaladat

**Affiliations:** 1 Department of Urology Keck School of Medicine University of Southern California Los Angeles California USA Institute of Urology and Catherine & Joseph Aresty Department of Urology, Keck School of Medicine, University of Southern California, Los Angeles, California, USA;; 2 Department of Urology University of Minnesota Minneapolis MN USA Department of Urology, University of Minnesota, Minneapolis, MN, USA

**Keywords:** Cystectomy, Urinary Bladder Neoplasms, Robotic Surgical Procedures, Neoadjuvant Therapy

## Abstract

**Purpose:**

To evaluate the perioperative mortality and contributing variables among patients who underwent radical cystectomy (RC) for bladder cancer in recent decades, with comparison between modern (after 2010) and premodern (before 2010) eras.

**Materials and Methods:**

Using our institutional review board-approved database, we reviewed the records of patients who underwent RC for primary urothelial bladder carcinoma with curative intent from January 2003 to December 2019. The primary and secondary outcomes were 90- and 30-day mortality. Univariate and multivariable logistic regression models were applied to assess the impact of perioperative variables on 90-day mortality.

**Results:**

A total of 2047 patients with a mean±SD age of 69.6±10.6 years were included. The 30- and 90-day mortality rates were 1.3% and 4.9%, respectively, and consistent during the past two decades. Among 100 deaths within 90 days, 18 occurred during index hospitalization. Infectious, pulmonary, and cardiac complications were the leading mortality causes. Multivariable analysis showed that age (Odds Ratio: OR 1.05), Charlson comorbidity index ≥ 2 (OR 1.82), blood transfusion (OR 1.95), and pathological node disease (OR 2.85) were independently associated with 90-day mortality. Nevertheless, the surgical approach and enhanced recovery protocols had no significant effect on 90-day mortality.

**Conclusion:**

The 90-day mortality for RC is approaching five percent, with infectious, pulmonary, and cardiac complications as the leading mortality causes. Older age, higher comorbidity, blood transfusion, and pathological lymph node involvement are independently associated with 90-day mortality.

## INTRODUCTION

Bladder cancer is among the top 10 most diagnosed cancers worldwide, with approximately 573,000 new cases and 213,000 deaths every year (1). It is also one of the most incident urological malignancies in the United States, with over 83,000 new cases and 17,200 deaths estimated in 2021 (2). The primary treatment for muscle-invasive and selected high-risk non-invasive urothelial bladder carcinoma (UBC) is radical cystectomy (RC) which is required in about a third of bladder cancer patients (3). RC remains to be one of the most complicated urological procedures, with a considerable rate of morbidity and postoperative complications (4, 5). There is a noteworthy rate of mortality for RC according to the National Cancer Data Base (NCDB), reporting the 30- and 90-day mortality rates to be 2.7% and 7.2% overall, and 1.9% and 5.7% in high-volume institutions, respectively (6).

Recent developments of minimally invasive surgical approaches, including robot-assisted radical cystectomies (RARC), as well as advancements in perioperative care, particularly enhanced recovery after surgery (ERAS) protocols, have been reported to improve the experience for patients undergoing RC (7, 8). The use of RARC has significantly increased, including up to a third of all RC cases in recent years. The robotic approach has been associated with a reduced risk of perioperative blood transfusion, complications, and length of hospital stay in older studies (9). However, some of the more recent randomized control trials and meta-analyses have reported no significant differences between open and RARCs in terms of complication rate and hospital stay (10). ERAS protocols are evidence-based multimodal pathways that include optimizations of pre-, intra-, and post-operative care to enhance the recovery following surgery. Our comprehensive institutional RC-ERAS protocol started off in 2012 (7) and evidenced a shorter hospital stay, yet no changes in readmission or early postoperative complications (11). Moreover, the use of neoadjuvant chemotherapy (NAC) for muscle-invasive bladder cancer has been gradually increasing, given its proven survival benefit from 7.6% in 2006 to 34.1% in 2014, according to the NCDB (12).

Most of the studies pertaining to perioperative mortality from RC are older studies and from the premodern era (before 2010), when the surgical interventions and perioperative management were performed traditionally (13, 14). Given the recent advancements in the management of bladder cancer, we aimed to assess the perioperative mortality rates as well as their contributing variables among patients undergoing RC in the modern era compared to those in the premodern era to better understand the impact of these improvements on patient outcomes.

## MATERIALS AND METHODS

### Patient Population and Management

Using our institutional review board-approved bladder cancer database (# HS-01B014), we retrospectively reviewed the records of consecutive patients who underwent RC for primary urothelial bladder carcinoma with curative intent from January 2003 to December 2019. Patients underwent RC and urinary diversion either by the robotic or open approach, depending on the surgeon’s and/or patient’s preference. All patients underwent extended pelvic lymphadenectomy given our institutional standards (14). The patients were also enrolled in our previously described enhanced recovery pathway during the modern era (7). The year of operation was classified as modern era (Jan 2010 to Dec 2019) and premodern era (Jan 2003 to Dec 2010).

### Data Variables

The demographic, clinical, pathological, and operative variables included the year of surgery, age, sex, Charlson comorbidity index (CCI), body mass index (BMI), use of neoadjuvant systemic therapy, surgical approach, type of urinary diversion, use of ERAS, estimated blood loss, transfusion, operative time, pathologic stage, histology, and positive margin.

The primary outcome variable of this study was 90-day mortality and the secondary outcome variables included 30-day mortality and the leading causes of death.

### Data Analysis

Associations between clinicopathological characteristics and outcomes were assessed by univariate models using Chi-squared and Wilcoxon rank sum tests for categorical and continuous variables, respectively. Logistic regression models were applied for multivariable analysis.

SAS Version 9.4 (SAS Institute Inc., Cary, NC, USA) was used for all data analyses. All p-values are 2-sided, and p < 0.05 was considered statistically significant.

## RESULTS

### Patients’ Features

A total of 2047 patients (1654 males and 393 females) with a mean(±SD) age of 69.6±10.6 years were included in this study. Open and robotic approaches were performed in 1666 (81.4%) and 381 (18.6%) patients, respectively. Among all patients, 469 (22.9%) had NAC, and 781 (38.2%) were enrolled in our institutional enhanced recovery pathway.

### Mortality Rates and Causes

The 30- and 90-day mortality rates were 1.3% and 4.9%, respectively. Moreover, during our study period, 90-day mortality rates were 5.3% (2003-2009) and 4.7% (2010-2019), and 30-day mortality rates were 0.9% (2003-2009) and 1.5% (2010-2019); however, these differences were not significant. Table-1 demonstrates the univariate analysis of the impact of perioperative variables on 90-day mortality.

**Table 1:** Univariate analysis of the impact of perioperative variables on 90-day mortality following radical cystectomy.

Variable	90-day Mortality (n=100)	Alive > 90 days (n=1947)	p-value
**Year of Surgery, n (%)**			
2003-2009	39 (5.3)	703 (94.7)	0.59
2010-2019	61 (4.7)	1244 (95.3)	
**Age (year), n (%)**			
≤ 65	13 (1.9)	661 (98.1)	**< 0.001**
> 65	87 (6.3)	1286 (93.7)	
**Gender, n (%)**			
Male	80 (4.8)	1574 (95.2)	0.80
Female	20 (5.1)	373 (94.9)	
**CCI, n (%)**			
0	23 (2.7)	817 (97.3)	**< 0.001**
1	18 (4)	437 (96)	
≥ 2	59 (7.8)	693 (92.2)	
**BMI, mean+SD (Kg/m^2^)**	26±5.8	27.5±5.1	0.006
**NAC, n (%)**			
No	80 (5.1)	1498 (94.9)	0.54
Yes	20 (4.3)	449 (95.7)	
**Surgical approach, n (%)**			
Robotic	21 (5.5)	360 (94.5)	0.51
Open	79 (4.7)	1587 (95.3)	
**Diversion, n (%)**			
Orthotopic	36 (2.9)	1216 (97.1)	**< 0.001**
Heterotopic	64 (8.1)	731 (91.9)	
**ERAS, n (%)**			
No	58 (4.6)	1208 (95.4)	0.46
Yes	42 (5.4)	739 (94.6)	
**Transfusion, n (%)**			
No	26 (2.7)	928 (97.3)	**< 0.001**
Yes	74 (6.8)	1019 (93.2)	
**Operative Time, mean+SD (hour)**	5.8±1.5	6.0±1.5	0.5
**Pathologic Stage, n (%)**			
≤ T2, N0	31 (2.6)	1176 (97.4)	**< 0.001**
> T2, N0	29 (7.5)	359 (92.5)	
Any T, N+	40 (8.8)	412 (91.2)	
**Histology, n (%)**			
Pure UC	77 (4.5)	1620 (95.5)	0.13
Variant	23 (6.6)	327 (93.4)	
**Positive margin, n (%)**			
No	86 (4.5)	1815 (95.5)	**0.01**
Yes	14 (9.6)	132 (90.4)	

CCI = Charlson Comorbidity Index; BMI = body mass index; NAC = neoadjuvant chemotherapy; UC = urothelial carcinoma; ERAS = enhanced recovery after surgery; EBL = estimated blood loss

Among the 100 deaths within 90 days, 18 (18%) occurred during index hospitalization, and 27 (27%) deaths happened within 30 days. The leading causes of 90-day mortality were determined to be infectious/septic, pulmonary, and cardiac complications (Table-2).

**Table 2:** Leading cause of death in 100 patients who died within 90 days following RC.

Type of complication (n)	Total number (%)
**Infectious/sepsis**	25 (25%)
Urinary tract infection (7)	
Bowel leak/fistula (6)	
Pneumonia (non-aspiration) (4)	
Sepsis of unknown origin (4)	
Small bowel obstruction/infection (3)	
Surgical site infection (1)	
**Pulmonary**	19 (19%)
Aspiration (±pneumonia) (8)	
Pulmonary embolus (6)	
Respiratory failure/ARDS (5)	
**Cardiac**	12 (12%)
Myocardial infarction (8)	
Cardiac arrythmia (4)	
**Disease progression**	5 (5%)
Brain metastasis (2)	
Peritoneal carcinomatosis (2)	
Liver and bone metastases (1)	
**Renal**	4 (4%)
Acute kidney injury (4)	
**Neurologic (including stroke)**	2 (2%)
**Gastrointestinal**	1 (1%)
Acute colonic pseudo-obstruction (1)	
**Unknown (including FTT)**	32 (32%)

ARDS = acute respiratory distress syndrome; FTT = failure to thrive

**Figure 1:**
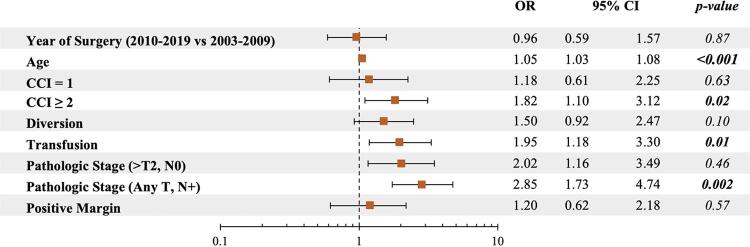
Forest plot for independent predictors of 90-day mortality in patients undergoing radical cystectomy.

On univariate analysis, higher CCI and heterotopic urinary diversion were associated with 30-day mortality. Given the small number of deaths within 30 days, a multivariable analysis was not performed. Independent predictors of 90-day mortality included age (Odds Ratio: OR 1.05, p < 0.001), CCI ≥ 2 (OR 1.82, p = 0.02), blood transfusion (OR 1.95, p = 0.01), and pathological nodal disease (OR 2.85, p = 0.002) (Figure-1). Although BMI, type of urinary diversion, and positive surgical margin showed statistical significance in univariate analysis of factors affecting the 90-day mortality (Table-1), they lost their significance in the multivariable model (Figure-1).

## DISCUSSION

In the current study, the perioperative mortality rate and its contributing variables for RC patients in the modern and premodern era (past two decades) in a tertiary referral center are compared. We found that the perioperative mortality rate following RC was consistent throughout the periods prior to and after 2010, despite increased use of neoadjuvant therapies, minimally invasive approaches, and implementation of enhanced recovery pathways. We report that infectious and pulmonary complications were the leading causes of mortality, while age (OR 1.05), Charlson comorbidity index ≥ 2 (OR 1.82), blood transfusion (OR 1.95), and pathological node disease (OR 2.85) were independent predictors of 90-day mortality.

The 30- and 90-day mortality rates have commonly been used as outcome measures for perioperative death for RC patients (13, 15). In our study, we found the 30- and 90-day mortality rates to be 1.3% and 4.9%, respectively, which was on par with the current literature on the contemporary cystectomy series (15, 16). During our study period, the mortality rates were consistent in the past two decades, too. A recent systematic review of 66 articles reports the weighted mortality rate to be 2.1% (0.0–3.7) for 30-day and 4.7% (0.0–7.0) for 90-day mortality following RC (16). Additionally, our 1.3% 30-day mortality rate over our study period (2003-2019) was comparable to the mortality rates in our institution for patients treated from 1971 to 2001, which was 2% (13). Historically, ever since the dramatic reduction in early surgical mortality rates for RC from 33% in the first cystectomy series in 1949 to 11% in the 1970s and 2.5% in 1978-1985, the postoperative survival rates have remained fairly consistent in the past few decades (13, 15, 16). Similar to our findings, Zhang et al. found no difference in 90-day mortality between patients with or without enhanced recovery following RC (17). While ERAS has been shown to improve postoperative recovery, other studies have confirmed that ERAS does not affect cancer-specific or overall survival (3, 18, 19). Moreover, our results showed that the surgical approach was not a predictor of 90-day mortality, which was on par with the current literature showing comparability for recurrence-free-, progression-free-, cancer-specific-, and overall-survival rates among open and RARC cohorts (20, 21).

In our study, infectious/sepsis, pulmonary, and cardiac complications were the leading causes of death which were consistent with Maibom et al.’s systematic review, containing 17 studies, that reports the leading causes of death to be 30% for cardiopulmonary events, 11% for sepsis, and 15% for bladder cancer progression (16). It should be noted that there is limited data on the leading causes of mortality following RC as larger databases such as SEER lack the details needed to determine the underlying cause of death, and most of the data comes from studies with a smaller sample size (22). Various studies have reported gastrointestinal and infectious to be common complications following RC (16, 23). The aforementioned systematic review also showed the weighted average complication to be 29.0% for GI, 26.4% for infectious, 5.0% for respiratory, and 6.1% for cardiac following RC (16). The previous report of 1,359 patients undergoing RC at our institution (1971-2001) reported cardiovascular and infectious/septic complications as primary and secondary causes of death (13). However, the current study demonstrated infectious followed by pulmonary and cardiovascular complications as the leading causes of early mortality following RC, respectively.

Like similar studies, our findings demonstrated age to be an independent predictor of perioperative mortality; in this study, older patients had about a 5% higher chance of 90-day mortality following RC with every additional year. Older age cohorts have consistently been reported to have a higher perioperative mortality rate (15, 24). A study of over 24,000 patients, using the National Cancer Database, also reported significantly increased postoperative mortality for older age groups (25).

In line with most of the current literature, our study showed that comorbidity status independently predicts postoperative mortality rates. Comorbidities have been reported to be strong predictors of cancer survival not just for UBC but for other types of cancer, either by increasing surgery-related complications, limiting treatment options, or directly leading to death (26).

Our study also showed blood transfusions to be a predictor of postoperative mortality, which is supported by the literature. Multiple studies report that perioperative transfusions are associated with an increased risk of mortality, however, there is still debate as to what degree this is due to blood transfusions serving as a marker for perioperative complications versus blood transfusion being an absolute risk for mortality (27).

This study also showed pathological lymph node involvement was associated with 90-day mortality by almost tripling the chances of 90-mortality. Other studies have confirmed the association of lymph node involvement with a higher rate of mortality for RC patients (28). It has been shown that the lower number of positive lymph nodes is a predictor of overall survival as well as disease-specific survival (29), while a study of over 2,000 patients found lymph-node involvement did not affect overall or cancer-specific survival (30). In a study reporting perioperative mortality of RC in our institution between 1971 and 2001, Quek et al. found no difference in blood transfusions or lymph node involvement between patients with and without 90-day mortality; this could be due to the limited number of patients who died post-surgery in the study period (13).

This study examined the mortality rates following RC and associated variables for our institution between 2003-2019. Some of the strengths of our study included having homogenous patients that had surgery in a similar fashion with the same-trained surgical team reducing surgeon bias. However, our study was not without limitations. Given the retrospective nature of our study and our sample size being confined to a single center, our results should be interpreted with caution. Despite reviewing a high volume of RC patients between 2003-2019, the number of patients who died within 30 or 90 days was still small. In addition, the primary cause of mortality was not accurately identifiable for a third of our patients who died within 90 days.

## CONCLUSIONS

Despite the recent advancements, the 30- and 90-day mortality rates following radical cystectomy have remained consistent in the past two decades. The 90-day mortality rate is close to 5%, while infectious/sepsis, pulmonary, and cardiac complications are the leading causes of death. The 90-day mortality was independently predicted by older age, higher comorbidity, blood transfusion, and pathological lymph node involvement.
